# Cytokine Networks between Innate Lymphoid Cells and Myeloid Cells

**DOI:** 10.3389/fimmu.2018.00191

**Published:** 2018-02-07

**Authors:** Arthur Mortha, Kyle Burrows

**Affiliations:** ^1^Department of Immunology, University of Toronto, Toronto, ON, Canada

**Keywords:** innate lymphoid cells, Group 1 ILCs, ILC2, ILC3, myeloid cells, macrophages, dendritic cells, cytokines

## Abstract

Innate lymphoid cells (ILCs) are an essential component of the innate immune system in vertebrates. They are developmentally rooted in the lymphoid lineage and can diverge into at least three transcriptionally distinct lineages. ILCs seed both lymphoid and non-lymphoid tissues and are locally self-maintained in tissue-resident pools. Tissue-resident ILCs execute important effector functions making them key regulator in tissue homeostasis, repair, remodeling, microbial defense, and anti-tumor immunity. Similar to T lymphocytes, ILCs possess only few sensory elements for the recognition of non-self and thus depend on extrinsic cellular sensory elements residing within the tissue. Myeloid cells, including mononuclear phagocytes (MNPs), are key sentinels of the tissue and are able to translate environmental cues into an effector profile that instructs lymphocyte responses. The adaptation of myeloid cells to the tissue state thus influences the effector program of ILCs and serves as an example of how environmental signals are integrated into the function of ILCs via a tissue-resident immune cell cross talks. This review summarizes our current knowledge on the role of myeloid cells in regulating ILC functions and discusses how feedback communication between ILCs and myeloid cells contribute to stabilize immune homeostasis in order to maintain the healthy state of an organ.

## Introduction

Maintaining a physical barrier to the external environment is vital to proper physiology and function of the body’s organs. However, environmental signals including nutrients, xenobiotic chemicals, microbial metabolites, together with cell intrinsic triggers like proliferation, cell death, damage, and metabolism play an essential role in influencing tissue homeostasis within organs. The immune system is a fundamental sensory system that contributes to physiology far beyond its classical role as modulator of immunity (1). Transient and long-lasting changes in the composition of these signals require appropriate interpretation by tissue-resident immune sensors, to ensure appropriate adjustments to environmental stimulation. Failure to achieve a balanced response through adequate instruction of non-sensory tissue-resident immune cells and incoming recruited immune cells resets the local tissue immune tone and ultimately affects the homeostatic physiology of an organ (2). While this description is generalized, it reflects many of the recent findings on tissue-resident elements of the immune system across lymphoid and non-lymphoid organs (3). The contribution of tissue-resident cells to organ homeostasis through sensory mechanisms and specialized effector functions sets the homeostatic tissue tone and is thus an essential process to preserve proper physiology of our body and its organs.

Innate lymphoid cells (ILCs) are a new family of innate immune cells composed of at least three independent lineages with distinct transcriptional profiles and effector functions. These cells predominantly reside in non-lymphoid tissues like the skin, lung, liver, intestine, and adipose tissue with organ-specific enrichment of different ILC subsets (4). Even though ILCs constitutively secrete cytokines and effector proteins, required to locally sustain tissue and immune homeostasis, their capacity to sense perturbations in the surrounding environment is limited (5, 6) (www.immgen.org). Interestingly, ILCs sparsely replenish their tissue-resident pool from circulating precursors but rather, self-maintain locally (7). ILCs thus resemble a tissue-resident immune cell type that executes local effector functions depending on the interpretation of the environmental state by an instructive sensor. However, there are two exceptions, the composition of the nutrient and metabolite pool in the immediate environment and secreted neuropeptides seams to be directly recognized by ILCs and influences ILC development and function during steady state and inflammation (8–12). The neuron–immune interaction through neuropeptides represents a new aspect of tissue-ILC crosstalk and environmental sensing. Even though this area of research is still in its infancy, it greatly advances our view on innate immune control by the neuronal network (13). Environmental metabolic components (i.e., amino acids, polyaromatic carbohydrates, vitamins, and lipids) control the ligand-activated transcription factors Aryl Hydrocarbon Receptor (Ahr) and members of the Retinoic-acid Receptor (Rar) family (14, 15). These mechanisms, although utilized by many cells, decorate ILCs with relevant direct sensory capacity impacting tissue development, protection, and homeostasis (16, 17). Although nutrient sensors like Ahr or Rar are widely expressed across the immune compartments and serve as sensor for metabolites, these receptors affect the development of several immune cells including ILCs (18). In addition, several non-dietary ligands are able to bind Ahr and result in its transcriptional activity or inhibition. These observations renders the actions of Ahr on its target cell highly dependent on environmental signals (19). Most strikingly, pathogen-associated Ahr ligands have been shown to enable the direct recognition of pathogens by Ahr-expressing ILCs and may thus be seen as ILC-intrinsic sensor of microbial ligands (17).

Myeloid cells are the earliest immune cells arising in our body and reflect a heterogeneous family of cells containing phagocytic and granulocytic cells. Members of these family either rapidly enter the tissue out of the blood circulation or self-maintain locally as tissue-resident patrolling sensors of the environment. Their migratory properties and excellent sensory machineries enable myeloid cells to recognize perturbations (i.e., cell death or infection) and immediately initiate a specific, local immune response (20, 21). Detailed genomic analysis of myeloid cells on high dimensional levels revealed tissue-specific epigenetic marks coupled to characteristic gene expression profiles depending on the local tissue environment (22–25). Interestingly, transfer of myeloid cells from one tissue to another will reprogram the myeloid gene expression profile, supporting the idea that myeloid cells are superior in recognizing and adapting to their local tissue environment (23). With ILCs being a tissue-resident cell type with limited capacity to recognize microbial signals, myeloid cells, as superior sensors of microbial and other tissue-derived signals, play a crucial role in controlling ILC homeostasis and function (26, 27). Acute and chronic activation of tissues is accompanied by changes in the local myeloid immune cell pool followed by adaptation of the tissue-resident ILC compartment (28–31). Our recent progress in understanding the tissue-specific distribution of myeloid cells and ILCs coupled to a detailed picture of their gene expression profile sheds light on the potential local dialog of these cells during health and disease. Current research is uncovering new pathways and cross talks that may be suitable to target these interactions with potential therapeutics, to reset diseased tissues to a healthy homeostatic state. Within this review, we highlight our current knowledge on the interactions of ILCs and myeloid cells, focusing on the cytokine-mediated cross talk of ILCs and myeloid cells.

## Diversity of Innate Lymphocytes

**Figure 1:**
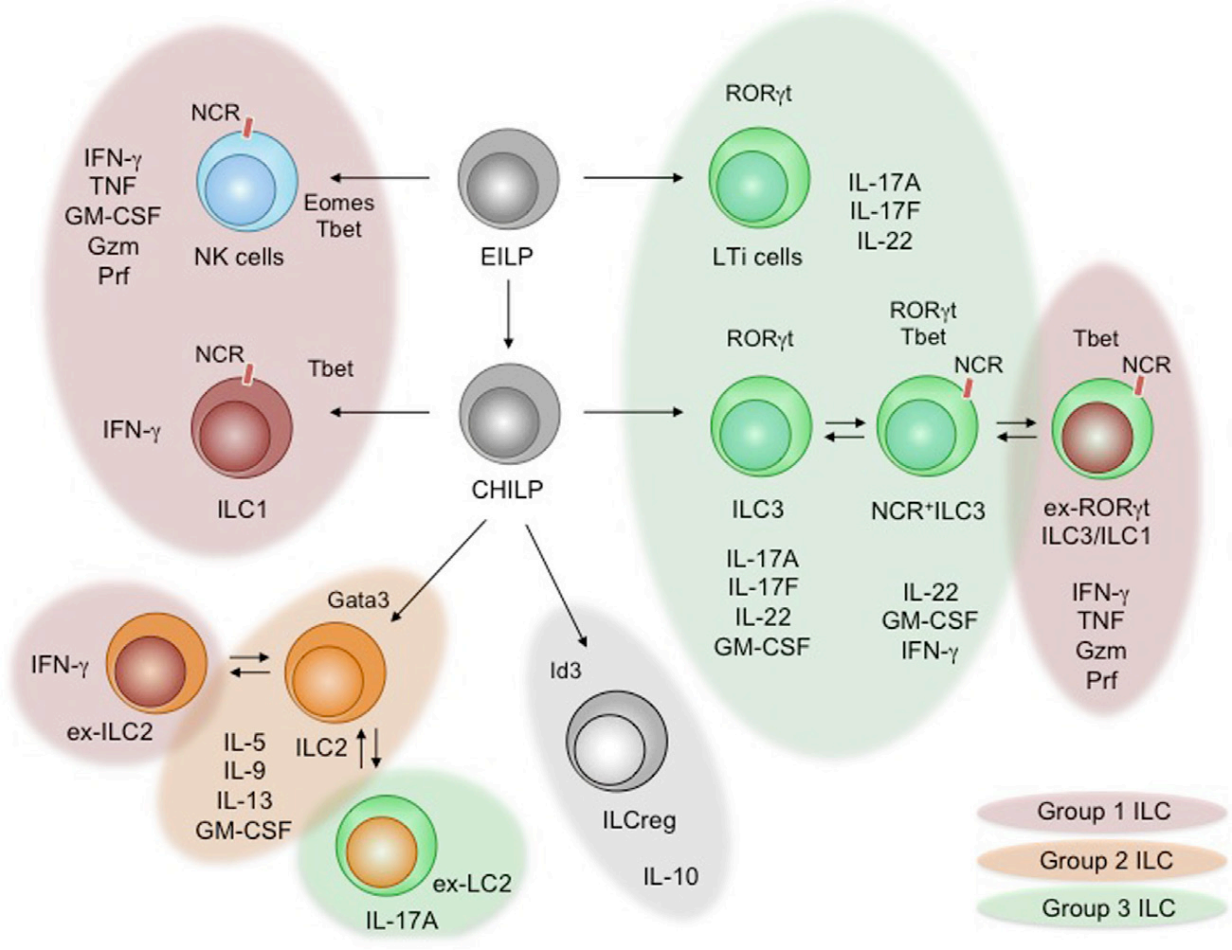
Development and functional specialization of innate lymphoid cells (ILCs). The diagram shows the developmental pathways of ILCs and branching into their different lineages. The *early innate lymphoid precursor* (EILP) and the *common helper innate lymphoid precursor* (CHILP) are progenitors to all ILCs (36, 37). Critical lineage-determining transcription factors are shown. Arising ILCs [natural killer (NK) cells, lymphoid tissue inducer (LT) cells, ILC1–3, and ILCreg] are displayed, including arising subsets. Individual groups of ILCs are indicated through color schemes. The expression of natural cytotoxicity receptors (NCRs) on ILC subsets is indicated. Specific and shared effector cytokines secreted during inflammation and steady state are listed below the indicated subsets of ILCs. Documented plasticity within group 2 ILCs and group 3 ILCs is indicated using overlapping and colored bubbles.

Unlike T and B cells, ILC-poiesis is independent of Recombination-activated gene (*Rag*) and *Rag*-dependent rearrangement of antigen receptors for their development but require signals through the common gamma chain (γ*_c_*). The cytokines thymic stromal lymphopoietin (TSLP), stromal cell-derived interleukin (IL)-7, and IL-15 also play dominant roles in their survival and homeostasis (32, 33). Concerted actions of these cytokines and others lead to the activation, priming and execution of effector functions of ILCs (34). The family of ILCs contains three lineages of ILCs (group 1 ILC, group 2 ILC, and group 3 ILC). These groups comprised natural killer (NK) cells (included in group 1 ILC), ILC1, ILC2, ILC3, and lymphoid tissue inducer (LTi) cells (included in group 3 ILC) that collectively mirror the effector profiles of most T helper (h) cells and cytotoxic T cell lineages (35) (summarized in Figure 1).

The development of all ILCs is tightly controlled by transcriptional programs that are shared with T cells, but additionally requires a unique composition of transcription factors to determine ILC commitment (e.g., Tox, Id2, Plzf, Nfil3, and Gata3) (38). In general, common innate lymphocyte precursors (CILPs) are lymphoid precursor cells in the adult bone marrow that are identified as Lin^−^ CD127^+^ Id2^+^ Nfil3^+^ Tox^+^ Plzf^high^ cells and have unrestricted potential to differentiate into all groups of ILCs (39). Common helper innate lymphocyte precursor (CHILP) is an immediate descendent of CILPs and characterized as Id2^high^ and Plzf^−^ cells, which retain the potential to exclusively give rise to ILC1, ILC2, and ILC3, while lacking the potential to differentiate into NK cells and LTi cells (36). NK cells arise from precursor NK cells that are generated from CILPs, while LTi cells arise early during ontogeny from a fetal liver lymphoid precursor sharing transcriptional homologies with ILC3 (36, 39, 40). Being unique in their developmental origin, CHILP-derived ILCs are separated into three lineages (ILC1, ILC2, and ILC3) characterized by lineage-specific transcription factors and effector functions that mirror Th1, Th2, and Th17 cells (6, 41–44). Observations in *Rag2^−/−^* mice identified lymphoid cells secreting cytokines commonly associated with Th1, Th2, or Th17 lineage commitment supporting the functional and developmental homology of ILCs and Th cells (45–48). During the steady state and even stronger during the onset of tissue inflammation, ILCs are a potent local source of cytokines that rapidly prime the “immunological tone” of a tissue (49). Given the limited capacity of ILCs to directly recognize tissue inflammation, the effector profile of these cells strikingly relies on cells interpreting the state of the tissue and communicating the presence of homeostasis, danger, or damage to ILCs.

Group 1 ILCs (ILC1) are identified as lin*^−^* NK1.1^+^ CD49b*^−^* KLRG1*^−^* IL-7R^+^ CD117*^−^* cells that secrete high levels of interferon (IFN)-γ and TNF-α but express little to no Granzyme (Gzm) or Perforin (Prf). Like Th1 cells, ILC1 are developmentally dependent on the T box transcription factor *Tbx21* (Tbet) and produce high amounts of their signature cytokine IFN-γ to protect from intracellular pathogens and contribute to chronic inflammatory pathologies (6, 36, 50, 51). Unlike NK cells that share an effector program with cytotoxic CD8^+^ T cells, ILC1 are independent of *Eomesodermin* (Eomes), originate from CHILP and lack the capacity to lyse target cells (27, 36). Based on their phenotypic similarity to NK cells and other ILC subsets (discussed below), group 1 ILCs might thus represent a heterogeneous population of innate effectors (51). The increasing appreciation of plastic behavior within ILC subsets may thus simply reflect the activation of a developmentally different and heterogeneous pool of ILCs within the ILC1-like features (Figure 1) (52).

Group 2 ILCs (ILC2) are identified as lin^−^ KLRG1^+^ IL-7R^+^ CD117^−^ IL33R^+^ IL1R2^+^. ILC2 represent an innate Th2 counterpart, which is developmentally tied to high expression of the transcription factor Gata-binding protein 3 (Gata3) and essential in the anti-parasite/helminth defense through the production of the cytokines IL-4, -5, -9, -13, and GM-CSF (6, 43, 51, 53, 54). ILC2 have further been associated with allergic reactions and tissue repair (55–57). Interestingly, different ILC2 subsets and activation states have been identified, enhancing the interest in understanding the contribution of different ILC2 subsets to defense, autoimmunity, or tissue repair (51). Changes in ILC2-specific cytokine secretion and adaptation of mixed effector phenotypes within ILC2 have been uncovered, adding functional plasticity to the ILC2 lineage (58, 59). Under chronic inflammatory conditions of the airways, ILC2 start to show features of ILC1 and express Tbet and IFN-γ (59–62). This shift toward an ILC1-like phenotype depends on the cytokines IL-1, IL-12, and IL-27 secreted by macrophages and dendritic cells (DCs) (63). Interestingly, adaptation of IL-17 production by ILC2 has been reported during Candida infection, suggesting a highly plastic behavior in order to adapt to the inflammatory context (64).

The last and currently most diverse group of ILCs matching a Th cell lineage, are group 3 ILCs (ILC3), including LTi cells. LTi cells (likely including ILC3 by the time of discovery) were first identified as a population of lymphocytes accumulating in the developing lymph nodes of embryos to initiate lymph node and lymphoid cluster formation through surface Lymphotoxin (sLTα_1β2_ and LTα_3_)–LTβ receptor interaction with stroma cells. *In vitro* cultures of purified LTi-like cells revealed that LTi-like cells are capable of expressing NK cell receptors and major histocompatibility complex (MHC) II on their surface, resembling features of NK cells and antigen-presenting cells (40). The NK cell receptor-expressing population was later identified as an independent subset of the ILC3 lineage (45, 46, 65–67). Recent reports confirmed the expression of MHCII^+^ ILCs, particularly on subsets of the ILC3 and ILC2 lineages, granting these ILCs with the ability to regulate adaptive T cell responses directly (68–70). LTi cells and ILC3 most closely mirror retinoic acid-induced orphan receptor (ROR)γt-dependent Th17 cells through their production of the cytokines IL-17A, IL-17F, IL-22, and GM-CSF (34). Importantly, ILC3s contain a population of RORγt^+^ cells, expressing natural cytotoxicity receptors (NCRs; i.e., NKp46, NKG2D, NK1.1), sharing transcriptional and functional features with NK cells and ILC1 (i.e., expression of Tbet, Gzm, Prf, IFN-γ and IL-12R) (71). These cells were termed NCR^+^ILC3 and led to the subdivision of ILC3s into NCR^−^ILC3s and NCR^+^ILC3s (72). The expression of the transcription factor RORγt in NCR^+^ILC3 does not define a stable end state and is repressed by the cytokines IL-12 and IL-15 (71, 73, 74). NCR^+^ILC3 that lost their expression of RORγt were identified using genetic fate mapping and conclusively termed ex-RORγt ILC3 (71). Like ILC1 these cells express Tbet, high levels of IFN-γ and are attributed with the capacity to contribute to colitis-like pathologies through the secretion of IFN-γ and GM-CSF (31, 50, 71, 75). The stage of ex-ILC3s, as a final/stable differentiation state, was challenged by a recent report demonstrating that ex-ILC3 are able to induce RORγt in the presence of IL-23 and retinoic acid (RA) potentially adapting their effector function to changes in the environmental cytokine milieu from inflammatory to homeostatic (75). Fate mapping of ILCs using NKp46-Cre mice reveals that NCR^+^ILC3 might actually downregulate the expression of NCRs to adopt an NCR^−^ILC3 phenotype (76). These routes of plasticity among ILC3s appear to be shifted from RORγt^+^ILC3 toward ex-RORγt ILC3 under chronic inflammatory conditions, suggesting environmental signals as regulator of ILC3 plasticity.

Natural killer cells are the oldest and most studied population of ILCs. They were identified as lymphocytes capable of lysing target cells in the absence of antigen-presentation, associated by the secretion of high amounts of the inflammatory cytokines IFN-γ, TNF-α and IL-2. NK cells are able to recognize classical self-MHCI and non-classical MHCI-like molecules, as well as NCR-ligands through activating or inhibiting NCRs during steady state and during stress (77). While the expression of activating and inhibitory NCRs allows the direct sensing of environmental states, the integration of these signals into NK cell functions heavily depends on the signaling module associated with the recognizing NCR (78). Activating NCRs allow NK cells to lyse and kill ligand expressing target cells and thus, unlike ILC1, share functional similarities with cytotoxic CD8^+^ T cells (79). The transcriptional regulators Tbet and Eomes are essential for NK cell development and function, including the production of IFN-γ and the execution of cytotoxicity, emphasizing their importance in anti-viral defense and anti-tumor immunity through direct cytotoxicity (80, 81). Interestingly, the exclusive role of NK cells in anti-tumor immunity was challenged by reports demonstrating a role for ILC3 and ex-ILC3 in contributing to potent anti-tumor immunity (82, 83).

Making up a highly diverse family of innate effector cells, ILCs execute a diverse range of homeostatic and inflammatory functions that are important for repair and defense of the body. Being of tissue-resident nature, ILCs permanently execute their effector functions locally driven by tissue-derived signals (26). Even though ILCs retain features of direct environmental sensing, their capability to recognize foreign microbial patterns remains poor and requires extrinsic sensory immune elements. Myeloid cells are perfectly equipped to undertake this task and play a dominant role in tissue homeostasis and inflammation by triggering repair and defense (84, 85). Communication between myeloid cells as sensors of the environment and ILCs as effector at the local tissue site is an attractive model accounting for early adaptations of organs to infections and resolution.

## Myeloid Cell Development

Myeloid cells across our body arise from clonogenic myeloid-primed precursors (MPPs) branching up into granulocytic or monocytic-primed progenitors giving either rise to polymorphonuclear leukocytes (PMNL) like basophils, mast cells, eosinophils and ganulocytes, as well as mononuclear phagocytes (MNPs) like monocytes, macrophages, DC, and plasmacytoid DC. The transcriptional circuits responsible for this diverse branching of the hematopoietic tree have been extensively reviewed elsewhere and will not be discussed in this review. However, myelopoiesis is one of the earliest processes during embryonic hematopoiesis, it continuously occurs throughout adulthood. While the yolk sac and the fetal liver are early sources for myelopoiesis, its primary source during adulthood is the bone marrow-resident precursor pool (85). The strongly inflamed tissue poses as an exception of this pattern, as undifferentiated myeloid precursors locally differentiating into mature myeloid cells have been reported in the acute and chronically inflamed spleen, lung. and intestine of mice (20, 86–88). Tissue-resident MNPs arise from early erythroid-myeloid precursors (EMP) in waves of myelopoiesis during ontogeny. These cells seed the developing organs and locally self-renew in the steady state, while chronic inflammation fosters the replacement of tissue-resident myeloid cells by progeny arising from circulating precursors (89, 90). MNPs including monocytes, macrophages, and DCs are generated from a common monocyte DC precursor (MDP) that requires cytokine-mediated signaling for its differentiation (84). A clonogenic progenitor with restricted potential to the monocyte/macrophage lineage has been described as Common Monocyte Precursor (cMoP) giving rise to both Ly6C^low^ and Ly6C^high^ monocytes, the latter being the immediate precursor to macrophages of the adult tissue. A recent report suggested a linear lineage positioning of cMoP upstream of Ly6C^hi^ monocytes that differentiate into Ly6C^lo^ monocytes following the fate to develop into a macrophage (91). The ontogeny of macrophages during different stages of development, including their transcriptional regulation has been reviewed excessively, is the subject of ongoing research and will not be the focus of the review (3). However, monocytes leaving the bone marrow into the circulation enter tissues for their terminal differentiation into macrophages. Recent epigenetic and transcriptomic characterizations of macrophages across almost the entire body demonstrated conserved commonalities within macrophages across all tissue but more interestingly identified substantial tissue-dependent heterogeneity within the enhancer landscape and the gene expression profile in a tissue-dependent fashion (22, 23). Monocytes as immediate precursors of macrophages do not display any of these tissue-dependent traits prior to local differentiation into macrophages, pointing at tissue-derived signal as key driver of terminal macrophage heterogeneity. On the contrary, single cell profiling of precursor DCs (preDCs) uncovered pre-committed subpopulations giving rise to the two major DC lineages termed cDC1 and cDC2 previously called CD103^+^ DC or CD11b^+^DC, before leaving the bone marrow to migrate into the circulation (24, 92). cDC1-primed and cDC2-primed preDCs then enter this tissues and fully differentiate into their mature progeny to adopt tissue-specific activation marker expression. Even though diverse activation stages within the tissue have been identified for terminally differentiated granulocytes, basophils, eosinophils, or mast cells, it is currently not known if tissue specification within PMNLs exists across different organs or within their early precursor population (93). Multiple-primed precursors (MMPs) with PMNL potential have been identified; their capacity to differentiate into multiple myeloid cell types renders this population rather uncommitted and heterogeneous (94). While eosinophils develop from an IL-5 receptor expressing population within the granulocyte-macrophage precursor (GMP) pool, basophils and mast cells develop from a common basophil-mast cell precursor, giving rise to further committed basophil or mast cell precursor cells (95, 96). Surprisingly, common routes of myeloid cell development can be short cut and located to non-bone marrow niches to generate MNPs and PMNLs under conditions of acute infection or chronic inflammation. This process, called “emergency granulopoiesis” or “extramedullary myelopoiesis”, rapidly generates large quantities of granulocytes, mast cells, basophils eosinophils, and monocytes depending on the type of inflammation. Reports identified the spleen and the intestine as prominent sources of extramedullary myelopoiesis (86, 88). Even though very few myeloid precursors are found in the steady state intestine or spleen, chronic autoimmune-driven intestinal inflammation or parasitic worm infections drive a substantial increase in these populations. Under these conditions, GMPs and MPPs give rise to granulocytes and macrophages, or basophils, eosinophils, and mast cells, respectively (86, 88). However, the contribution of bone marrow versus extramedullary myelopoiesis during steady state is currently unknown.

## Induction of Feedback Communication Between Tissue-Resident ILCs and Myeloid Cells

Within diverse microenvironments and through selective precursor populations, myeloid cell development displays a highly complex and heterogeneous process adapted to the physiology of the hosting organ. With functions far beyond immunity, myeloid cells execute processes that are tailored to serve the physiology and homeostasis of the organ. Thus, functional diversification of myeloid cells, adapting to the local milieu adds another layer of complexity to the processes of myeloid specialization. ILCs are a potential tissue determinant of myeloid diversification as their tissue-resident production of cytokines, upon stimulation, can act on myeloid cells to reprogram their effector profile. However, with myeloid cells being the prominent tissue-resident sensors of the tissue environment, their activation precedes the activation of ILCs. Thus, it is important to first understand myeloid-derived signals as potent drivers of ILC function in order to ultimately determine the effects of cytokine-mediated feedback responses by ILCs on myeloid cells. These tissue-specific innate immune cell cross talks reprogram the local cytokine milieu and set the immune tone for tissue infiltrating immune cells prior to the initiation of an adaptive immune responses.

## MNP-Derived Stimuli

**Figure 2:**
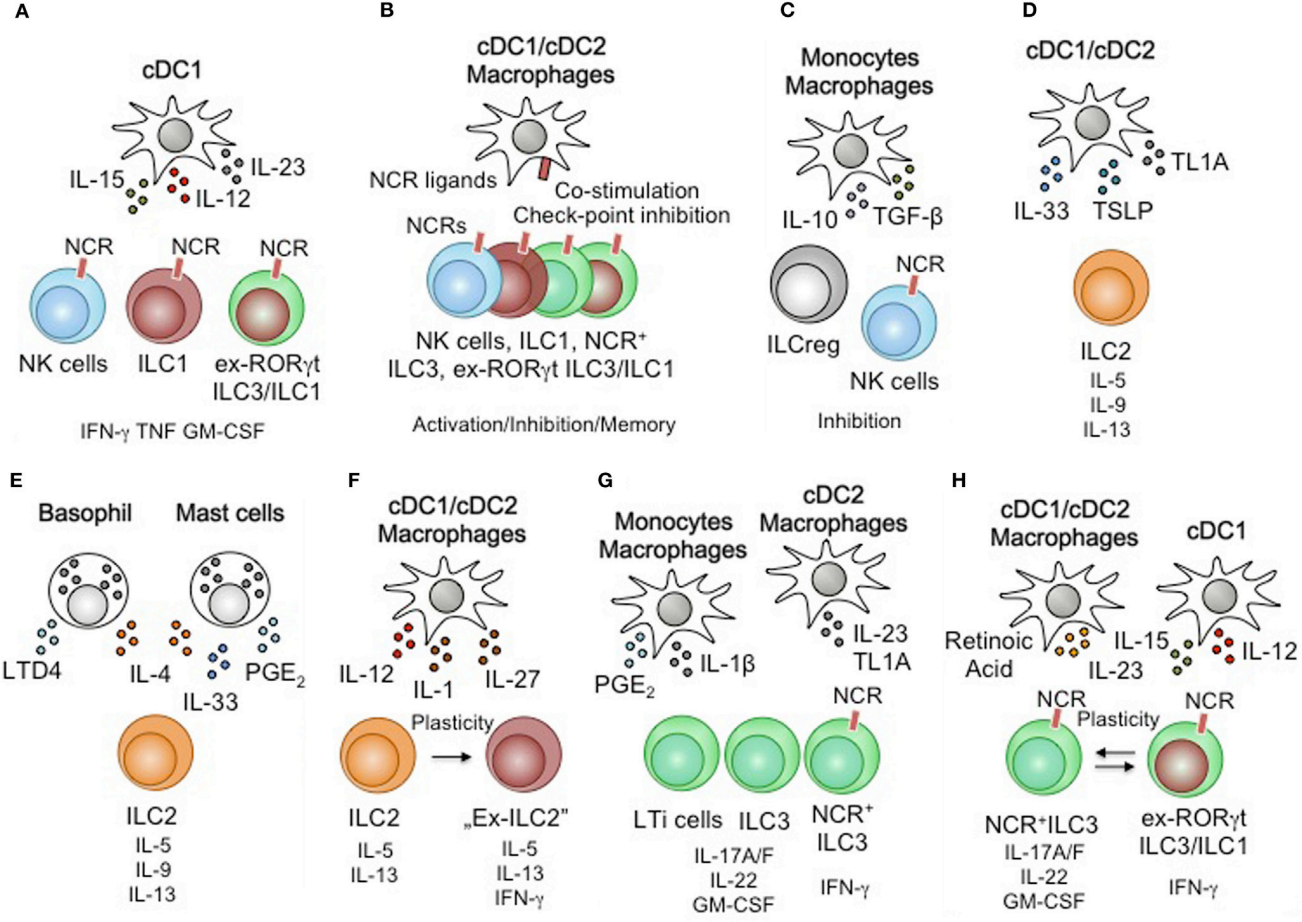
Myeloid regulation of innate lymphoid cells (ILCs) function and plasticity. **(A)** Dendritic cells (DCs) activate natural killer (NK) cells, group 1 ILCs (ILC1), ex-ILC3/ILC1 through interleukin (IL)-12, IL-15, and IL-23. **(B)** Natural cytotoxicity receptor (NCR)-expressing ILC subsets are subject to receptor-ligand mediated activation or inhibition. Improved immune responses through re-occurring engagement of activating receptors have been associated with memory-like behavior in NK cells. Co-stimulatory receptors and checkpoint inhibitory ligands could control the interactions of macrophages/DCs and ILCs. **(C)** Monocyte/macrophages inhibit NK cells via IL-10 and TGF-β secretion. **(D)** DC subsets are capable of activating ILC2 through the production of IL-33, thymic stromal lymphopoietin (TSLP), and TL1A. **(E)** PMN leukocytes (basophils and mast cells) activate ILC2 through IL-33, IL-4, and lipid mediators. **(F)** DC subsets and macrophages influence the stability of ILC2 by inducing type 1 immune features through IL-12, IL-1, and IL-27 leading to plastic behavior of ILC2. **(G)** ILC3 are activated by monocyte/macrophages and DCs. Lipid mediators and the cytokines IL-1β, IL-23, and TL1A drive ILC3 effector functions. **(H)** Retinoic acid, IL-23, IL-15, and IL-12 are myeloid-derived cytokines that control the plasticity and transition of NCR^+^ILC3 and ex-RORγtILC3/ILC1.

Monocytes, macrophages, and DCs express highly conserved sensory machineries enabling them to recognize danger signals, pathogens, and foreign antigens (i.e., malignant cells, infected cells, bacteria, parasites, and viruses) to initiate protective immunity (97, 98). Toll-like receptors, NOD-like receptors, lectins, and Fc-receptors are intra- and extracellular sensors that trigger the activation of myeloid cells to alert the local cellular microenvironment. MNPs utilize the secretion of the cytokines IL-1α, IL-1β, IL-33, and IL-18 in order to achieve this goal (97, 98). As epithelial cells are also able to produce IL-1α, IL-18, and IL-33, tissue-resident non-myeloid sensors also contribute to the pool of cytokines to activate tissue-resident immune cells. However, as the sensory machinery of epithelial cells and MNPs differs, these different sensory compartments may be poised to react to distinct and defined stimuli. Monocytes, macrophages, and DCs secrete the cytokines IL-12, IL-15, TL1A, and IL-23 upon engagement of pattern recognition receptors and are able to synergistically amplify cytokine signaling in ILCs by IL-1α, IL-1β, IL-33, and IL-18 (26, 71, 99–101) (Figure 2A). Collectively, IL-1α, IL-1β, IL-12, IL-15, TL1A, IL-18, IL-23, and IL-33 are MNP-derived, locally produced cytokines that allow rapid stimulation of tissue-resident ILCs and thus determine the local immune tissue-tone upon danger recognition (Figures 2A,B). However, similar to the communication of DCs and T cells through MHC-peptide-antigen receptor complexes, MNPs including DCs are able to express MHC-like surface receptors that engage activating and inhibiting NCRs on ILC subsets (102, 103) (Figure 2C). These receptor-ligand interactions modulate the tissue environment and are an essential element in fine-tuning ILC-responsiveness in the steady state as well as during infection and cancer (104). Noteworthy, MNPs are metabolically active cells able to catabolize lipids, vitamins and amino acids, all of which are implicated in controlling the development and function of different ILC subsets (11, 105). With metabolites affecting ILC function and development, MNPs may modulate tissue-specific immunity by dampening and inhibiting the activation of ILC subsets through their contribution of specific metabolites (Figure 2D). MNPs further secrete the cytokines IL-10 and TGF-β, both immunologic inhibitors. Similar to inhibitory NCR-MHC-like receptor-ligand interactions, anti-inflammatory cytokines are potential negative regulators of ILCs arising from MNPs (106). MNPs thus collectively provide activating and inhibiting signals through cytokines, NCR-ligands and metabolites to control the activation of tissue-resident ILCs. With recent findings in mice, identifying an Id3-dependent ILC subset that secrets IL-10 and TGF-β, such inhibitory signals could also arise internally from within the ILC pool (107).

## PMNL-Derived Stimuli

Basophils, eosinophils, mast cells, and neutrophils are PMNL that either reside locally within tissues or rapidly enter tissues from the circulation. PMNL are equipped to recognize tissue damage, parasitic worm infections and antigen-antibody complexes of the IgE isotype thus differing significantly from MNP (108, 109). PMNL contain cytosolic granules loaded with mediators of inflammation (e.g., eicosanoids and prostaglandins). Within minutes after activation, PMNL release these mediators, histamine, serotonin, proteases, reactive oxygen species, and anti-microbial peptides from their preloaded cytosolic granules followed by a delayed wave of secreted cytokines and chemokines to recruit additional myeloid cells. The immediate response of PMNLs in the tissue, similar to the response of MNPs, changes the local cytokine milieu and activates surrounding tissue-resident innate immune cells. PMNL therefore sets the local immune tone through activating signals that differ from MNPs (95, 110). Much like their tissue-resident MNP counterpart, basophils and mast cells are locally residing cells that adapt a tissue-specific transcriptional profile in the steady state (93, 111). IL-4 and IL-33 are prominent cytokines released by activated basophils and mast cells that are potent activators of ILCs (112) (Figure 2E). Eosinophils and neutrophils infiltrate injured tissues out of the circulation, initiate a local antimicrobial immune response, and serve as an amplifier of mast cell and basophil-induced tissue inflammation. Eosinophils and neutrophils do not respond to mast cell and basophil-derived cytokines but require stimulation by additional cytokines. PMNL are producers of prostaglandine E2 and leukotrine D4 both lipid mediators and potent drives of ILC activation (113–115) (Figure 2E). PMNLs are thus tissue-resident or infiltrating myeloid cells controlling the local cytokines production by other tissue-resident immune cells like ILCs. This activation results in a feedback crosstalk of infiltrating myeloid cells and locally activated ILCs.

## Myeloid Activation of ILCs

Tissue-resident and infiltrating myeloid cells utilize diverse sensory machineries to integrate disturbances of the tissue into the activation of local immune cells. This is achieved through the secretion of cytokines, the release of lipid mediators and activating NCR-ligands. While other non-hematopoietic activators of tissue-resident immune cells have been identified (e.g., neuron, glial cells, fibroblasts, and epithelial cells) (10, 12, 34, 116), their sensory machinery and location may differ from myeloid cells and thus allow for the integration of more complex stimuli. In addition to activation, myeloid cells are able to inhibit tissue-resident immunity through cytokines and inhibitory NCR ligands. With ILCs being tissue-resident cells, these mechanisms control their local, tissue-specific activation, development, and function. Myeloid cells and other sensory non-hematopoietic cells are thus potent regulators of ILCs depending on their sensory receptors and downstream effector mechanisms.

Innate lymphoid cells are a diverse group of cells with characteristic transcriptional profiles determining their lineage commitment, differentiation, and function (35). Their effector machinery is primed to rapidly secrete large amounts of cytokines following antigen-independent stimulation by tissue-resident sensors. With myeloid cells being elementary in interpreting and integrating the local tissue state, MNP-derived or PMNL-derived stimulation selectively activates subsets of ILCs to determine the appropriate amplification of the tissue immune response. Type 1, 2 and 3 immune responses are tailored to defend an organ from specific pathogens. Type 1 combats viruses, intracellular bacteria, intracellular parasites and malignant cells. Type 2 protects against parasitic worm infections, while type 3 defends against fungal and extracellular bacterial attacks (117). The transcriptional programs of ILC1, ILC2 and ILC3s are tailored to selectively support these antimicrobial immune responses, respectively. The activation of either of these ILC subsets, however, is determined by signals arising from specific myeloid cells based on their cytokine receptor profiles (118).

## Type 1 Immune Responses and Myeloid Activation of ILC1, NK Cells, and ex-ILC3

Natural killer cells, ILC1, and ex-ILC3 are capable of receiving activating signals by myeloid cells through cytokine receptors. These ILCs express IL-12Rb1, IL-12Rb2, IL-2Rb, and IL-18R and transduce signals upon engagement by IL-12, IL-15, and IL-18 (6) (Figure 2A). Stimulation via these cytokines initiates the production of INF-γ, TNF-α, and GM-CSF and triggers a Type 1 immune response for the defense against intracellular bacteria, intracellular parasites, viruses, and tumors, but can also be attributed to the development of autoimmune pathologies like inflammatory bowel disease (34). Myeloid cells are capable of activating group 1 ILCs beyond secreted cytokines. The expression activating NCRs (e.g. NKp46, NKG2D, NKG2A and Ly49s) on NK cells, ILC1 and ex-ILC3 offer a receptor-ligand-mediated mechanism for ILC activation (41, 50, 71) (Figure 2B). Engagement of activating NCRs can drive cytotoxicity in NK cells and trigger the release of cytokines by ILC1 and ex-ILC3. Direct cell–cell contact of ILCs and myeloid cells thus contributes to an increase in effector function. Astonishingly, “memory-like” NK cells expressing the receptor Ly49H have been found to sustain their activation state and exhibit accelerated responses upon reengagement of this receptor (119). With recent observations describing the expression of PD1 on ILC3 subsets and additional inhibitory receptors being expressed by NK cells and ILC2 subsets (e.g., Ly49A, KLRG1, or CTLA4), a critical role of check point inhibitors and their ligands on myeloid cells requires future investigations (120–122). Noteworthy, two recent reports identified regulatory functions in ILC subsets suggest ILC-mediated negative regulations, particularly during inflammation (107, 123). Comparable to observations made in NK cells, ILC subsets can be inhibited by the suppressive cytokines IL-10 and TGF-β secreted by macrophages and a recently identified set of regulatory ILCs (106, 107) (Figure 2C). These suppressive activities are exclusively observed under conditions of strong or chronic inflammation. It thus remains to be shown if suppression of ILCs by ILCs or macrophages takes place during the steady state in order to maintain homeostasis.

## Type 2 Immune Responses and Myeloid Activation of ILC2

Group 2 ILCs express the cytokine receptors IL-2Ra, IL-4Ra, IL-9R, IL-17Rb, IL-1Rl1, and TSLPR and low levels of death receptor (DR)3, IL-1R2, and IL-12Rb1. These receptors render ILC2 susceptible to signaling through the cytokines IL-2, IL-4, IL-9, IL-25, TL1A, IL-33, and IL-12 (6, 34). These cytokines are produced by activated basophils, mast cells, cDCs, and macrophages and induced the release of GM-CSF, IL-4, IL-5, IL-9, and IL-13 by ILC2 (Figures 2D,E). Triggering the expression of IL-5, IL-9, and IL-13 is critical for the defense against parasitic worms and respiratory flu infections (47, 88). Noteworthy, exacerbated secretion of IL-5, IL-9, and IL-13 has been reported to be a driver of allergic reactions and chronic inflammatory conditions of the lung and skin (56, 124). More strikingly, mast cell and eosinophil-derived lipid mediators are strong non-cytokine-related drivers of ILC2-specific cytokine production (112, 113, 115) (Figure 2E). Conversely, the cytokines IFN-α, IFN-β, and IL-27 have been reported to inhibit ILC2 activity. Although the exact source of these cytokines within the hematopoietic compartment remains to be identified, it is anticipated to stem from myeloid cells (58). Concerted actions of inhibitory and activating cytokines, atypical to type 2 immune responses, revealed plastic adaptions of the secreted cytokine profile by ILC2 (59–61) (Figure 2F). Nonetheless, tissue-derived inhibition of ILC2 through the engagement of KLRG1 by E-cadherin further suggests ligand receptor-mediated suppression to dampen and control ILC2 activity (125).

## Type 3 Immune Responses and Myeloid Activation of ILC3

Group 3 ILCs express the cytokine receptors IL-1R1, IL-2Ra, IL-2Rb, IL-12Rb1, IL-12Rb2, IL-18R, IL-23R, DR3, and IFN-γR. ILC3 are thus responsive to IL-1, IL-2, IL-12, IL-15, IL-23, IL-18, TL1A, and IFN-γ. Stimulation through these receptors drives the production of IL-22, IL-17A, IL-17F GM-CSF, TNF-α, and IFN-γ (6, 34) (Figure 2G). These ILC3-derived cytokines play an important role in the defense against extracellular bacteria and fungi (26, 34, 126). Expression of ILC3-produced cytokines also supports tissue and immune homeostasis during the steady state through induction of immunosuppressive functions of DC and macrophages as well as the modification of local and systemic antibody responses (26, 116, 127). More strikingly, exacerbated activation of ILC3 has been implicated in chronic inflammatory diseases like inflammatory bowel disease (86, 128, 129). The activation of ILC3 thus requires contextual interpretation to fine tune their secreted signature cytokines to ensure homeostasis and prevent chronic inflammation. A mechanism negatively regulating ILC3 functions includes the epithelial cell-derived cytokine IL-25 (130). IL-25 is highly produced by Tuft cells, a crucial stimulus to activate ILC2 and essential to control parasitic worm infections (131). Understanding the exact molecular pathways to facilitate ILC3 inhibition requires more detailed future investigations. Interestingly, cytokine-independent myeloid activation of ILC3s represents a way of communication between NCR^+^ILC3 and their tissue-resident myeloid partners. NCR^+^ILC3 express the activating NCR NKp44. Crosslinking this receptor increases the capacity of NCR^+^ILC3 to secrete cytokines and induces a functional switch from homeostatic toward pro-inflammatory phenotype with an increase in INF-γ and TNF-α production (102) (Figures 2B,H). In line with these observations, enrichment of NKG2D-expressing ILC3s has been observed in psoriatric lesions of patients, suggesting that the expression of NKG2D ligands in the skin leads to the activation of skin-resident ILC3 (132). While these findings suggest receptor-ligand signaling through activating NCRs to stimulate ILC3s, the role of inhibitor NCRs on these cells remains enigmatic. In light of growing efforts to block inhibitory receptors on immune cells, to revitalize their effector program, receptor-ligand interactions controlling ILC3s are of great interest in order to therapeutically target ILC3s during chronic inflammatory diseases.

## Type 1 Immune Control through the ILC1/NK Cell/ex-ILC3–Myeloid Cell Axis

**Figure 3:**
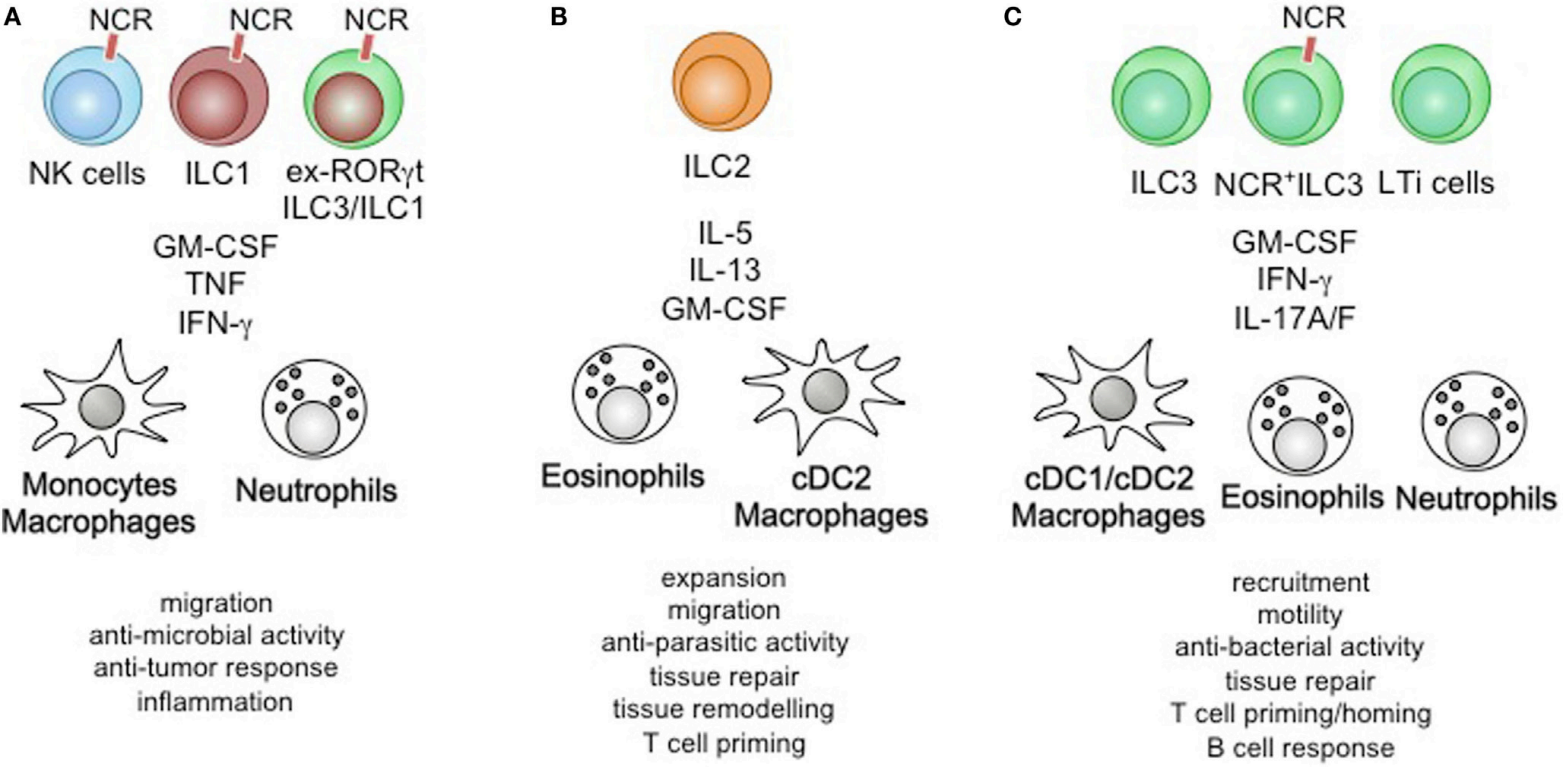
Summary of ILC-mediated myeloid cell activation on immunity and physiology. **(A)** Natural killer (NK) cells, Group 1 ILCs (ILC1), and ex-RORγt ILC3/ILC1 control the migration of monocyte/macrophages and neutrophils, modulate their anti-microbial activity and anti-tumor response leading to sustained inflammation. **(B)** ILC2-derived cytokines act on eosinophils, dendritic cells (DCs), and macrophages to control their proliferation, migratory behavior, role in tissue remodeling and repair. Furthermore, ILC2-mediated activation of myeloid cells is essential in anti-parasitic immunity and optimization of Th2 priming. **(C)** ILC3s control the recruitment, motility, and tissue repair through the activation of DC/macrophages, eosinophils, and neutrophils. The anti-bacterial immunity driven by ILC3-produced cytokines contributes to host-defense, while homeostatic execution of ILC3-specific effector functions improves T cell priming and innate B cell responses.

Group 1 ILCs, NK cells, and ex-ILC3 produce high levels of INF-γ, TNF-α, and GM-CSF. Neutrophils, monocytes, macrophages, and DC express INF-γR1, IFN-γRII, TNFR and the GM-CSF-specific receptor α and common β chain to integrate stimulation of these cytokines into their differentiation and maturation (97, 98) (Figure 3A). Each of these cytokines is able to individually activate monocytes, macrophages and DC through their corresponding receptor, resulting in specific effector functions like phagocytosis, antigen-presentation, lysosome degradation and the production of reactive oxygen species (133). Interestingly, stimulation by GM-CSF is used to drive the differentiation of monocytes into macrophages and DC, while synergistic activation through concerted actions of GM-CSF, IFN-γ, and TNF-α results in strong activation and inflammatory phenotypes (134). Recent findings demonstrated that GM-CSF and TNF-α play important role during the steady state by controlling myeloid cell homeostasis (135, 136). The steady state production of these cytokines thus controls the local myeloid cell population. The absence of GM-CSF, IFN-γ, or TNF-α renders mice highly susceptible to infections by viruses, intracellular bacteria, and intracellular parasites particularly at mucosal surfaces. While steady state expression of GM-CSF and TNF-α controls the local myeloid homeostasis, concerted actions of additional inflammatory cytokines (i.e., IFN-γ) or changes in the locally produced concentrations of GM-CSF and TNF-α could contribute to the development of tissue inflammation. The myeloid cell-induced production of IFN-γ, TNF-α and GM-CSF by ILC1, NK cells, and ex-ILC3 are thus essential feedback signals required to initiate antimicrobial immunity and inflammation, while steady state sources of these cytokines might control homeostatic functions of myeloid cells (42). ILC1 and ex-ILC3 have been found to accumulate and exacerbate chronic intestinal inflammation- driven by IL-12, IL-23, and IL-15 (41, 50, 71, 75, 86). In these studies, RORγt-expressing ILC3 lost their RORγt-controlled effector functions and adapted an ILC1-like phenotype associate with increased levels of secreted IFN-γ, which promote a pro-inflammatory phenotype in neutrophils and monocytes resulting in tissue damage and destruction. Furthermore, ILC-derived inflammatory cytokines promoted the secretion of matrix metalloproteases ultimately destabilizing the epithelial barrier (137). High levels of IFN-γ and GM-CSF influence the local myeloid cell population, but additionally induce changes in infiltrating myeloid cells and the local epithelial barrier. Changes in the location of myeloid cells within the inflamed tissue are associated with adaptations in myeloid cell recruitment and differentiation of myeloid precursor (86, 138). The effector functions of ILC1/NK cells and ex-ILC3 during inflammation therefore control the local and peripheral development of myeloid cells leading to a reprogramming of the local tissue-resident myeloid effector pool followed by an increased myeloid cell output by bone-marrow myelopoiesis and extramedullary granulopoiesis. Collectively, the activation of ILC1, NK cells, and ex-ILC3 by myeloid cells drives strong tissue inflammation and potentiates the pro-inflammatory type 1 immune response.

## Type 2 Immune Control through the ILC2-Basophil/Eosinophil/Mast Cell Axis

Group 2 ILCs are potent producer of IL-4, IL-5, IL-13, IL-9, and GM-CSF. ILC2 secreted cytokines are able to activate myeloid cells through the common β chain, IL-5Rα chain, IL-9Rα chain, IL-13R1, IL-13R2, common γ chain, and GM-CSFR α chain. Mast cells, basophils, eosinophils, macrophages, and DC express these receptors and are potential target cells for ILC2-derived cytokines (93, 97, 98) (Figure 3B). While IL-4, IL-9, IL-33, IL-25, and TSLP act on ILC2 to drive the secretion of IL-4, IL-5, IL-13, IL-9, and GM-CSF, a few of these cytokines act in an autocrine fashion on ILC2 to sustain the secretion of cytokines—being an important example of a positive feedback loop (34). IL-5 and GM-CSF are essential differentiation and activation factors for MPPs in the bone marrow and mucosal tissue. ILC2-produced cytokines were found to be key drivers of MPP-derived progeny cell differentiation (i.e., basophils, mast cells, eosinophils, and macrophages) upon stimulation by IL-25 and IL-33 and contributed to the local anti-parasitic host defense by enlarging the pool of tissue-resident myeloid cells through extramedullary myelopoiesis (87, 88). Interestingly, synergistic activity of IL-5 and IL-13 or IL-5 and IFN-γ was shown to modulate the phenotype of eosinophils, suggesting plastic behavior of eosinophils in the context of ILC activation (139). Concerted actions of INF-γ and IL-33 on ILC2-specific cytokine production revealed a similar pattern of adaptation to environmental cytokines by ILC2 (140). ILC2-derived IL-13 has been shown to acts on mast cells, macrophages, and DCs (34). In conjunction with other myeloid growth and differentiation factors like GM-CSF, IL-4 and IL-13 promote an alternatively activated macrophage phenotype that is essential in controlling adipocyte differentiation, lung tissue fibrosis, and anti-helmith immunity (141–143). DC exposed to the ILC2-produced cytokines IL-4 and IL-13 are more prone to licensing a type 2 Th cell response and support the recruitment of memory T helper cells to the inflamed airways during allergy (144). ILC2-derived cytokines further play a significant role in maintaining tissue macrophages through the recruitment of monocyte and the initiation of the differentiation into alternatively activated macrophages. More strikingly, ILC2-produced IL-13 is a critical factor driving allergic reactions and could be an interesting target for future therapeutic manipulation. Collectively, ILC2-derived cytokines control extramedullary myelopoiesis and specific adaptations of tissue-resident myeloid cells in the context of type 2 immune responses.

## Type 3 Immune Control through the ILC3–Monocyte/DC/Granulocyte Axis

Group 3 ILCs express the Th17-associated cytokines IL-17A, IL-17F IL-22, and GM-CSF in the steady state and IFN-γ during inflammation (6, 34). These cytokines engage the IL-17R, IFN-γR, and the GM-CSFR on myeloid cells. Macrophages, neutrophils, eosinophils, and DC are particularly responsive to GM-CSF even though the expression level of the GM-CSF-specific GM-CSFR alpha chain varies among these cells (23, 97) (Figure 3C). Much like other cytokines expressed by ILC3 (i.e., IL-22 and IL-17A), GM-CSF production requires instructive signal from the intestinal microbiota commonly sensed by myeloid cells and epithelial cells (26). ILC3-derived GM-CSF has been implicated in connecting the innate and adaptive immune response through its actions on myeloid cells. It imprints macrophages, DC and neutrophils with the capacity to produce RA, IL-10, and BAFF, cytokines that contribute to the generation of regulatory T cells, gut homing of T cell, and the production of innate antibody responses in the steady state (26, 71, 126, 127). Under inflammatory conditions, GM-CSF acts in concert with IFN-γ, TNF-α, and pathogen associated molecules to induce a switch in effector functions and polarization (134). It controls the anti-microbial activity of neutrophils and eosinophils and contributes to exacerbated inflammation at mucosal tissues (128, 137). Most striking, both the absence of and the chronic production of GM-CSF result in high susceptibility to intestinal pathology, demonstrating the importance of its balanced expression and activity to maintain homeostasis (145, 146). Additionally, promiscuous expression of GM-CSF at tissue sites that commonly show little to no expression of GM-CSF has been associated with an increased pathology (147). These findings suggest that increased expression of ILC3-derived GM-CSF or ectopic expression in concert with other cytokines support inflammation. It will be important to dissect the role high versus low level of GM-CSF expression in the context of tissue inflammation, as well as the localized actions on the myeloid effector pool. With some reports demonstrating IFN-γ production in ILC3, the concerted expression of GM-CSF and IFN-γ by ILC3 could help to identify unique inflammatory ILC3 subsets that would be an interesting target for treatment of chronic inflammatory conditions.

## Discussion

Research over the last decade extended our understanding of ILCs as a new and exciting cell type within the innate immune system. ILCs are a potent source of homeostatic cytokines in the steady state and essential in tuning inflammatory innate and adaptive immune responses (4). Being members of the tissue-resident immune compartment, ILCs respond to cytokines released by activated myeloid cells in order to sustain their own cytokine profile. Several ILC-derived cytokines signal back to myeloid cells and in turn control their survival, recruitment, functional profile or developmental origin. The communications of ILC subsets and their matching myeloid counterpart within the tissue are elementary to early host defense and significantly contribute to chronic inflammatory processes like IBD, psoriasis, multiple sclerosis, obesity, or asthma. Deciphering the essential homeostatic and inflammatory cytokines, their modes of orchestrated action within the network of ILC-regulating cytokines will greatly advance our understanding of how the myeloid cell–ILC axis drives defense or triggers chronic inflammation. New *in vitro* cell culture systems will be instrumental in characterizing the responsive signaling pathways within ILCs to identify selective drug targets (148–150). With ILC-derived cytokines acting on myeloid cells and their precursors, functional and developmentally distinct myeloid cells arise at sites of tissue-resident ILC activation. Identifying the contribution of individual cytokines to the onset of this myeloid reprogramming will uncover the role specific ILC-derived cytokines during defense or chronic inflammation and should be another priority to target ILCs for tissue-specific modulation. While ILCs can secrete several cytokines at the same time, subsets of ILCs at the site of activation could collaborate to confer more then two cytokine-signals to myeloid cells. The analysis of ILC subsets and their cytokine profiles during health and disease will help identify inflammatory cytokine-producing ILC subsets that either initiate defense, or chronic inflammation. A systematic approach is required to understand myeloid-derived cytokine networks that control the crosstalk of myeloid cells and ILCs in order to tailor interventions of their communication. Understanding the changes of ILC and myeloid cell-derived cytokines at the local tissue site during, steady state, infection or chronic inflammation will allow the design of strategies to revert tissue-specific changes in innate immune activation associated with inflammation. Several clinical trials targeting inflammatory cytokines are in pre-clinical or clinical phase, aiming to block tissue-specific inflammation by systemic neutralization of cytokines. While successful therapies are based on this approach of cytokine neutralization (e.g., Infliximab or Anakindra), other targeting strategies resulted in severe side effects that exacerbated the pathology (151). The cytokine-mediated cross talks described in this review offer several targets that could be utilized to intervene in the myeloid cell–ILC communication. Targeting approaches however, should take into account that fine-tuning the myeloid cell-ILC axis, rather then blocking it completely might be desirable. Sustained, homeostatic activation of ILCs should be maintained during targeting approaches. With strong developmental and functional homology between mouse and human ILCs across tissues, animal models are an attractive system to screen for myeloid cell–ILC interactions that could be translated into the human setting. In summary, understanding the cytokine-mediated cross talks between myeloid cells and tissue-resident ILCs could lead to a better understanding of tissue homeostasis and chronic tissue inflammation.

## Author Contributions

AM and KB wrote the manuscript and designed figures.

## Conflict of Interest Statement

The authors declare that the research was conducted in the absence of any commercial or financial relationships that could be construed as a potential conflict of interest.
